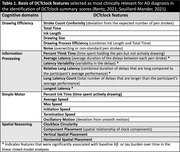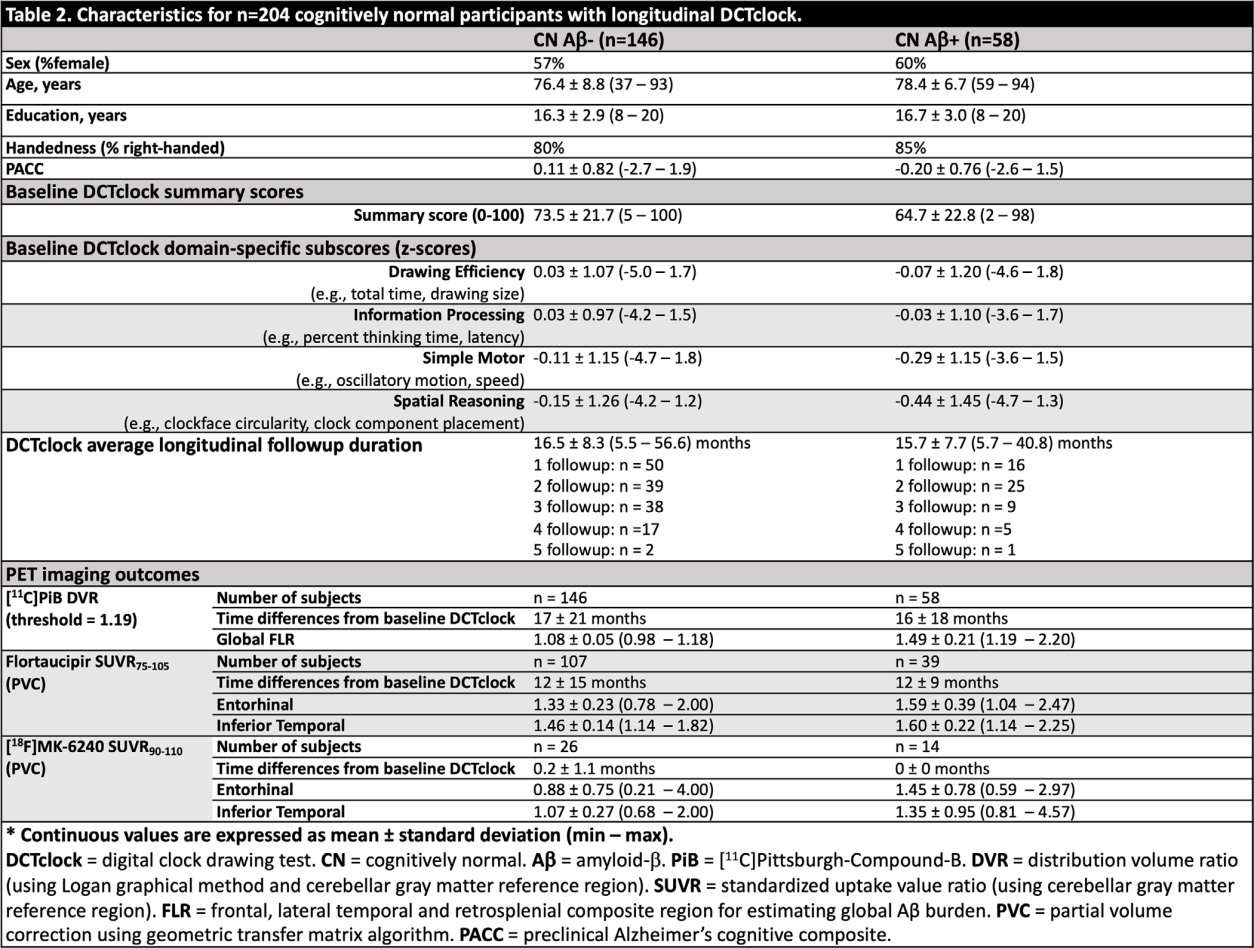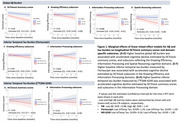# Elevated amyloid‐ß and tau burden is associated with accelerated cognitive decline on a digital clock drawing test in preclinical AD

**DOI:** 10.1002/alz.089175

**Published:** 2025-01-03

**Authors:** Jessie Fanglu Fu, Talia L Robinson, Marina Rodriguez Alonso, Adela Francis Malave, Roos J Jutten, Brian Healy, Grace A Del Carmen Montenegro, Emma G Thibault, Dana Penney, Randall Davis, Reisa A Sperling, Keith A Johnson, Julie C Price, Dorene M Rentz

**Affiliations:** ^1^ Massachusetts General Hospital, Boston, MA USA; ^2^ Massachusetts General Hospital, Harvard Medical School, Boston, MA USA; ^3^ Gordon Center for Medical Imaging, Massachusetts General Hospital, Boston, MA USA; ^4^ Lahey Hospital and Medical Center, Lexington, MA USA; ^5^ MIT Computer Science And Artificial Intelligence Laboratory, Cambridge, MA USA

## Abstract

**Background:**

PET quantifies tau and amyloid‐ß (Aß) pathology in preclinical AD. A 2‐min digital clock‐drawing test (**DCTclock^TM^**) captures clock‐drawing outcomes and processes, potentially more sensitive to cognitive deficits in preclinical AD than pencil‐and‐paper tests. The DCTclock summary score comprised subscores targeting multi‐domain cognitive performance (i.e., Drawing Efficiency, Information Processing, Spatial Reasoning, and Simple Motor). These subscores included unique features reflecting hesitation (pen‐stroke latencies) and speed during the drawing process (**Table.1**). Previous cross‐sectional study showed worse performance in the Spatial Reasoning domain was associated with high Aß and tau burden in older cognitive normal (**CN**) participants. *This study evaluates whether longitudinal changes in DCTclock performance are associated with Aß and tau burden in preclinical AD, and explores DCTclock features that are most important for such associations*.

**Methods:**

A total of 204 CN participants underwent baseline and follow‐up DCTclock assessments, Aß PET ([^11^C]PiB) and tau PET (Flortaucipir or [^18^F]MK‐6240, **Table.2**). Global Aß and regional tau burden were estimated as distribution volume ratios in neocortical aggregate regions and standardized uptake value ratios in entorhinal and inferior temporal using cerebellar gray reference, respectively. Separate linear mixed models examined associations between longitudinal DCTclock and 1) Aß, 2) tau, and 3) Aß and tau burden, adjusted for age, sex, and education. Separate models first examined DCTclock summary scores and domain‐specific subscores. Exploratory analyses then evaluated features within domains showing significant associations.

**Results:**

Elevated baseline Aß or tau is most strongly associated with accelerated decline in DCTclock performance in the Information Processing domain (**Fig.1**). Evaluating both Aß and tau in a single model, only tau showed significant associations with Information Processing subscores. The associations between DCTclock and Aß or tau were driven by latency features (Information Processing domain), including latency variability, longest latency, and average latency (**Table.1**). Notably, these features showed improved performance longitudinally in participants without Aß/tau burden, suggesting expected practice effects.

**Conclusion:**

Our results suggest that longitudinal decline in DCTclock performance, especially in information processing or executive function, is related to early Aß/tau burden in older CN adults. Diminished longitudinal DCTclock performance, especially in latency features uniquely captured by digital tools, may reflect greater cognitive efforts in preclinical AD.